# Public preferences and priorities for end-of-life care in Kenya: a population-based street survey

**DOI:** 10.1186/1472-684X-13-4

**Published:** 2014-02-15

**Authors:** Julia Downing, Barbara Gomes, Nancy Gikaara, Grace Munene, Barbara A Daveson, Richard A Powell, Faith N Mwangi-Powell, Irene J Higginson, Richard Harding

**Affiliations:** 1 Makerere University, PO Box 72518, Kampala, Uganda; 2 Formerly African Palliative Care Association, Kampala, Uganda; 3 Department of Palliative Care, Policy and Rehabilitation, Cicely Saunders Institute, King’s College London, Denmark Hill, London, UK; 4 Formerly African Palliative Care Association, Nairobi, Kenya

**Keywords:** Public health, Hospices, Palliative care, Attitude to death, Public opinion, Africa

## Abstract

**Background:**

End-of-life care needs are great in Africa due to the burden of disease. This study aimed to explore public preferences and priorities for end-of-life care in Nairobi, Kenya.

**Methods:**

Population-based street survey of Kenyans aged ≥18; researchers approached every 10th person, alternating men and women. Structured interviews investigated quality vs. quantity of life, care priorities, preferences for information, decision-making, place of death (most and least favourite) and focus of care in a hypothetical scenario of serious illness with <1 year to live. Descriptive analysis examined variations.

**Results:**

201 individuals were interviewed (100 women) representing 17 tribes (n = 90 44.8%, Kikuyu). 56.7% (n = 114) said they would always like to be told if they had limited time left. The majority (n = 121, 61.4%) preferred quality of life over quantity i.e. extending life (n = 47, 23.9%). Keeping a positive attitude and ensuring relatives/friends were not worried were prioritised above having pain/discomfort relieved. The three most concerning problems were pain (45.8%), family burden (34.8%) and personal psychological distress (29.8%). Home was both the most (51.1% n = 98) and least (23.7% n = 44) preferred place of death.

**Conclusion:**

This first population-based survey on preferences and priorities for end-of-life care in Africa revealed that psycho-social domains were of greatest importance to the public, but also identified variations that require further exploration. If citizens’ preferences and priorities are to be met, the development of end-of-life care services to deliver preferences in Kenya should ensure an holistic model of palliative care responsive to individual preferences across care settings including at home.

## Background

Palliative care improves the quality of life of patients and families who face life-threatening illness, by providing pain and symptom relief, spiritual and psychosocial support from diagnosis to the end of life and bereavement [1]. Although an estimated 60% of those dying annually would benefit from palliative care [2], globally there is great unmet need. The World Health Organization recommends a public health strategy for palliative care development [3], with key foundation measures, such as policy and education, needing to be premised on local need and evidence [4,5]. However, worldwide there is increasing recognition of the need to develop end-of-life and palliative care in response to evidence of people’s needs and wishes [6]. This pertains in Africa, too, where levels of unmet need for this kind of care are particularly high [7,8].

The development of palliative care is seen as an urgent humanitarian need, [7,9] and can be supported through existing global human rights legislation [10]. However, there is a relative lack of evidence within Africa of individuals’ preferences and priorities for end-of-life and palliative care [4,5]. Existing cross-sectional surveys and qualitative studies on end-of-life care preferences have been conducted mainly in the West [11-18]. Public polls undertaken in the USA, Australia and Europe have highlighted disparities between what people report they would like and what they actually receive at the end-of-life, with individuals in high income countries generally wanting to die at home [19-24]. In a recent study looking at preferences for place of death if faced with advanced cancer, in seven European countries (England, Flanders, Germany, Italy, the Netherlands, Portugal and Spain) between 51% (Portugal) and 84% (The Netherlands) would like to die at home. Personal values, along with age, were recognised as key influences on the preferred place of death [22]. This is in contrast to patients’ preferences and priorities for end-of-life care reported in Rwanda for people living with HIV and AIDS, with 67% of respondents stating that they would prefer to be cared for in hospital at the end-of-life. However, this study was undertaken in the specific context of a country affected by genocide and civil war, where participants reported that they did not have anyone to look after them at home, as many of their family members were lost during the genocide [25]. Studies looking at the palliative care needs of individuals in Uganda and Kenya, however, support the findings from the European study, suggesting that patients would prefer to be cared for at home [7,26-28]. This links in with the importance and value placed on psychosocial and spiritual issues, such as feeling at peace and having a sense of meaning in life, over physical issues within this context [29]. In light of the differing availability of end-of-life care, cultural perspectives, prevailing diseases, gross domestic products (GDP) and other issues such as a lack of trained health workers, overstretched health systems etc., data on preferences from high income settings cannot be extrapolated to low income countries.

In order to provide the necessary evidence to develop culturally appropriate, responsive end-of-life care, this study aimed to determine public preferences and priorities for end-of-life care in Nairobi, Kenya. The study was undertaken as part of an international collaborative project (PRISMA) to coordinate end-of-life care research and practice [30].

## Methods

### Study design and setting

A population-based street survey methodology was used, based on the methods of an associated pan-European telephone survey on public preferences and priorities for end-life-care [22]. The random-digit-dialling sampling method used in the European arm of the study was not thought to be appropriate. The sensitive nature of the topic meant that it would be important to establish a rapport between the person administering the survey and the respondent, and this is easier to do face-to-face. Death and dying is not a subject generally discussed within the Kenyan context and so, following discussion, it was felt that respondents were more likely to participate in the survey if it was administered face-to-face rather than on the telephone, thus ensuring someone was there to provide support if the respondents became upset. Whilst random-digit telephone surveys are common in the developed world, they are not common within countries such as Kenya, where many people do not have access to a telephone, with the distribution of phones being uneven [31]. Initial inquires were made with regards to a possible telephone survey but no company was identified that were prepared to undertake a survey of this nature. Therefore, we developed a novel street survey design, building on house-to-house and census based surveys that have been conducted successfully in Kenya and other parts of Africa.

The survey was conducted on 17 streets around the centre and western parts of the city of Nairobi. Streets were chosen with careful consideration of local knowledge of the city aiming to recruit participants from a variety of settings and backgrounds (e.g. cognisant of factors such as flow of people, researchers’ safety, areas used mainly by nationals). A variety of commercial and business districts were chosen and the timing of data collection coincided with the times when the streets would be busy but not so crowded that it would not be possible to collect data. To validate our methodology a pilot study with 17 participants was conducted, which demonstrated the methods to be feasible and acceptable with no issues regarding item sensitivity and cultural inappropriateness [32].

### Sampling and data collection

Two researchers jointly conducted data collection for safety reasons. They approached every 10th person, alternating between men and women, until they recruited at least 200 participants. Inclusion criteria were participants aged ≥18, Kenyan nationals and those able to understand and speak English (the language of the survey). A distress protocol was developed where researchers acknowledged the emotional nature of the topic, gave opportunity for stopping the interview and, if required, participants were given contacts for counselling and support. If the researchers were particularly concerned about a participant, they would discuss this with their manager to proceed with appropriate action to support the participant in question. The researchers administered a structured questionnaire (described below), reading the questions aloud to participants and recording their responses by hand. Following a description of the study, participants were asked if they have any questions and were also asked to give oral consent to participate in the study. If consent was not given, then the researcher did not proceed with the survey.

### Survey questionnaire

A version of the PRISMA European survey questionnaire was adapted and used [22,23,33]. Minor adaptations were made to ensure that it was appropriate for the street survey format in the African context. These adaptations included defining a hospice or palliative care unit as these are not so well known within Kenya and also included areas identified in the pilot study [32], such as working in pairs for safety.

The Kenyan questionnaire was structured into four main sections. The first concerned socio-demographics (age [actual], employment status [Employed/Not employed], education [Did not attend school, did not complete primary, completed primary, secondary, bachelors degree, post graduate degree], area urbanisation [urban, peri-urban, rural], province [list of provinces in Kenya], religion [open text] and tribe [open text]) along with participants’ experience of serious illness, death and dying (themselves or among their friends/family in the past five years, and/or caring for dying friends or family members). The remaining three sections contained questions regarding preferences and priorities for end-of-life care (Table 1). Participants were asked to consider a hypothetical scenario: “if you had a serious illness, for example cancer, and were likely to have less than one year to live…”. They were then asked about; a) their ‘life’ priorities i.e. aspects that would matter most to them if they were faced with the scenario presented; b) their preferences and priorities for care (for provision of information, involvement in decision-making, place of death, focus of care on quality or quantity of life); and, c) the symptoms and problems that would concern them the most at the end-of-life.

**Table 1:** Survey questions on preferences and priorities for end-of-life care

	
**a. ‘Life’ priorities at the end-of-life**	• When people are faced with a serious illness like cancer with limited time to live, they may have to make difficult decisions and prioritise some things over others. In this situation, how would you order the following four aspects by their level of importance to you, the first being the most important (1) and the last being the least important (4): keeping a positive attitude; having pain and discomfort relieved; having practical matters resolved; making sure relatives and friends are not worried or distressed?
**b. Care: preferences, priorities and focus**	• If you had a serious illness, for example cancer, and were likely to have less than one year to live, would you like to be informed that you had limited time left?
	• Would you like to be informed about what symptoms you were likely to experience?
	• Would you like to be informed about the options available for care and how they might effect you? These options might be services available, places where you could be looked after, treatments and medication.
	• Keeping in mind a situation of serious illness with less than one year to live, please consider that you were able to make decisions. Who would you like to make decisions about your care? yourself; your spouse or partner; other relatives; friends; the doctor; other.
	• What if you had lost your ability to make decisions, who would you most like to make decisions about your care? yourself (by specifying wishes before losing ability – for example in a living will; your spouse or partner; other relatives; friends; the doctor; other.
	• In a situation of serious illness, like cancer with less than one year to live, where do you think you would prefer to die if circumstances allowed you to choose? And where would you least want to die: in your own home; in the home of a relative or friend; in a hospice or palliative care unit (I.e. places with specialist care and beds for dying patients); in a hospital; in a nursing home; somewhere else?
	• What would matter most to you in the care available? Please choose from the following three aspects the one that would matter most to you: having as much information as you want; choosing who makes decisions about your care; dying in the place you want? And in second place?
	• When people are faced with a serious illness like cancer with limited time to live, they may have to make difficult decisions and prioritise some things over others. In this situation, would it be more important to extend your life or to improve the quality of life for the time you had left: to extend life; to improve the quality of life; both are equally important; you don’t know?
**c. Most concerning symptoms and problems**	• Which of the following nine symptoms or problems you think would concern you the most: having no energy; being in pain; changes in the way you look; having no appetite at all; being a burden to others; being unable to get your breath; being alone; feeling as if you want to be sick; being worried and distressed?

### Data analysis

The data were entered into an Excel spreadsheet and exported to SPSS v18 for analysis. All data were double-entered and cross-checked with the original paper questionnaire (discordances were evident in 43/54 variables, >4% in only five variables). Missing data were found in 15/54 variables (>2.5% in only three variables). Descriptive analyses examined variations in preferences and priorities for end-of-life care including life priorities, care preference priorities and focus, and most concerning symptoms and problems, an issue of particular relevance in Africa where access to medications such as strong opioids is often limited or non-existent [34-36].

### Ethical approval

Ethical approval was gained from the Kenya Medical Research Institute (KEMRI RES 7/3/1) and the ethics committee of PRISMA’s academic coordinating centre in Europe, King’s College London (BDM/08/09-100).

## Results

### Socio-demographics and experience of serious illness, death and dying

During the two-week period, 201 people (100 women) completed the survey (see Table 2). Mean age was 27 years. Half of the respondents were not in paid employment. Four percent (n = 8) reported having been seriously ill in the past five years and 42.6% (n = 84) had cared for a close relative or friend in their last months of life.

**Table 2:** Characteristics of participants (N = 201)

	
**Gender**	
*Female*	100 (49.8%)
*Male*	101 (50.2%)
**Education level**	
*Did not attend school*	2 (1.0%)
*Primary education*	18 (9.0%)
*Secondary education*	160 (79.6%)
*Completed bachelors degree*	21 (10.4%)
**Religion**	
*Christian*	195 (97%)
*Muslim*	4 (2.0%)
*African religion*	1 (0.5%)
*Jehovah’s witness*	1 (0.5%
**Urbanisation**	
*Urban*	143 (71.1%)
*Peri-urban*	44 (21.9%)
*Rural*	14 (7.0%)
**Province where they were born**	
*Central*	66 (33.2%)
*Nairobi*	31 (15.6%)
*Western*	29 (14.6%)
*Rift Valley*	28 (14.1%)
*Eastern*	26 (13.1%)
*Nyanza*	16 (8.0%)
*Coast*	3 (1.5%)
**Tribe to which they belong**	
*Kikuyu*	90 (44.8%)
*Luhya*	36 (17.9%)
*Kamba*	18 (9.0%)
*Kisii*	16 (8.0%)
*Kalenjin*	14 (7.0%)
*Meru*	11 (5.5%)
*Luo*	5 (2.5%)
*Embu*	3 (1.5%)
*Maasai*	2 (1.0%)
*Digo*	1 (0.5%)
*Giriana*	1 (0.5%)
*Mbeere*	1 (0.5%)
*Nandi*	1 (0.5%)
*Nubian*	1 (0.5%)
*Turkana*	1 (0.5%)
**Experience of death and dying**	
Close relative of friend died in the last five years	153 (77.3%)
Close relative or friend diagnosed with serious illness in last five years	88 (44.4%)
Supported and cared for close relative or friend in last months of life	84 (42.6%)
Personally diagnosed with serious illness in last five years	8 (4.0%)

### ‘Life’ priorities

Participants were asked how they would order four priorities if faced with a serious illness like cancer with limited time to live (see Table 3). A score of zero was given to the aspect considered least important, up to a score of three to that considered most important. Keeping a positive attitude (mean = 2.17), and making sure relatives and friends are not worried or distressed (mean = 1.46) were rated above having pain and discomfort relieved (mean = 1.22) or having practical matters resolved (mean = 1.14) (Table 3). Descriptive analysis showed no significant differences in the importance attributed to having pain and discomfort relieved, except for the effect of employment: more important to people who had a job than for those who were not employed (mean = 1.38 and 1.06, respectively).

**Table 3:** ‘Life’ priorities at the end-of-life

	**Mean scores**	**Sum scores**	**1st place N (%)**	**2nd place N (%)**	**3rd place N (%)**	**4th place N (%)**
**Keeping a positive attitude**	**2.17**	**434**	**103 (51.5%)**	**46 (23.0%)**	**33 (16.5%)**	**18 (9.0%)**
**Making sure relatives and friends are not worried or distressed**	**1.46**	**291**	**41 (20.6%)**	**67 (33.7%)**	**34 (17.1%)**	**57 (28.6%)**
**Having pain and discomfort relieved**	**1.22**	**242**	**33 (16.6%)**	**40 (20.1%)**	**63 (31.7%)**	**63 (31.7%)**
**Having practical matters resolved**	**1.14**	**227**	**22 (10.9%)**	**47 (23.4%)**	**67 (33.7%)**	**63 (31.7%)**

Footnote: A score of zero was given if considered least important, up to a score of three if considered most important.

### ‘Care’: preferences, priorities and focus

Asked if they were faced with a serious illness with less than one year to live, 96% (n = 193) responded they would always like to be informed about the symptoms they could experience. However, fewer (56.7% n = 114) said they would always like to be informed they had limited time left, with 9.0% (n = 18) only wanting to be told if asked, and 34.3% (n = 69) saying they would not want to be informed.

When respondents were asked whether they would like to make decisions about their care, 47.8% (n = 96) said yes, if they had the capacity to do so; however, more said they would like a relative to be involved (55.7% n = 112). Additionally, 45.8% (n = 92) wanted the doctor to be involved. If they did not have the capacity to make decisions, 68.2% (n = 137) of the respondents said they would want a relative to be involved, with 29.4% (n = 59) wanting the doctor involved (Table 4).

**Table 4:** Decision making and preferred place of care

**Who to involve in decisions about care at the end-of-life**	**When have mental capacity to make a decision N yes (%)**	**When have lost the mental capacity to make a decision N yes (%)**
Relative (partner, spouse or other relative)	112 (55.7%)	137 (68.2%)
Self	96 (47.8%)	15 (7.5%)
Doctor	92 (45.8%)	59 (29.4%)
Friend	9 (4.5%)	14 (7.0%)
Other	5 (2.5%)	5 (2.5%)
**Preferred place of death**	**Most preferred**	**Least preferred**
In your own home	98 (51.1%)	44 (23.7%)
In the home of a relative or friend	3 (1.6%)	39 (21.0%)
In hospital - but not palliative care unit	47 (23.5%)	35 (18.8%)
In a hospice or palliative care unit (I.e. places with specialist care and beds for dying patients)	30 (15.6%)	9 (4.8%)
In a nursing home	4 (2.1%)	7 (3.8%)
In a residential home	0	9 (4.5%)
Somewhere else	10 (5.2%)	43 (21.4%)

One’s own home was the most common preferred place to die (51.1% n = 98), but also the least preferred (23.7% n = 44). Hospital was the second most preferred place (23.5% n = 47) and the home of a relative or friend was the second most common least preferred place to die, with a similar proportion to those who said ‘own home’ (21.0% n = 39) (Table 4).

Having as much information as you want (mean =1.52) was prioritised above choosing who makes decisions about care (mean =1.06) and dying in the place you want to (mean =0.42) (Table 5). Regarding what respondents wished to be the focus of care if they had a serious illness like cancer with <1 year to live, 61.4% (n = 121) said improving quality of life, 23.9% (n = 47) said extending life, and 9.1% (n = 18) said both aspects were equally important.

**Table 5:** Priorities amongst three aspects of care (i.e., What would matter most to you in the care available?)

	**Mean scores**	**Sum scores**	**1st most important N (%)**	**2nd most important N (%)**	**3rd most important N (%)**
Having as much information as you want	1.52	300	124 (62.9%)	52 (26.4%)	21 (10.7%)
Choosing who makes decisions about care	1.06	208	54 (27.4%)	100 (49.8%)	43 (21.8%)
Dying in the place you want	0.42	83	19 (9.7%)	45 (23.0%)	132 (67.3%)

Footnote: A score of zero was given to the aspect considered least important, up to a score of two to the one considered most important.

### Most concerning symptoms and problems

Participants were asked which of nine problems or symptoms would concern them most. Being in pain, a burden to others and worried and distressed were rated as the most concerning (Table 6) and being in pain, a burden to others and being unable to get their breath as the second most concerning. Socio- demographic differences were seen with some symptoms, for example being in pain was more concerning with increased education level, changes in the way you look was more concerning for those who had experience in caring for a friend or relative, and feeling as if you want to be sick was more concerning for men than women.

**Table 6:** Ranking of concerning symptoms and problems at the end-of-life

	**Mean scores**	**Sum scores**	**1st place n (%)**	**2nd place n (%)**
Being in pain	1.74	350	57 (28.4%)	35 (17.4%)
Being a burden to others	1.53	307	36 (17.9%)	34 (16.9%)
Being worried and distressed	1.46	293	32 (15.9%)	28 (13.9%)
Being unable to get your breath	1.39	279	23 (11.4%)	32 (15.9%)
Being alone	1.25	252	16 (8.0%)	19 (9.5%)
Having no energy	1.24	249	15 (7.5%)	17 (9.0%)
Having no appetite	1.20	241	13 (6.5%)	14 (7.0%)
Changes in the way you look	1.13	228	6 (3.0%)	15 (7.5%)
Feeling as you want to be sick	1.05	212	3 (1.5%)	5 (2.5%)

Footnote: A score of three was given to the most concerning and a score of two to the second most concerning (all other problems and symptoms were given a score of one).

## Discussion

This is the first population-based survey of public preferences and priorities for end-of-life care in Africa. We found people placed optimism (through keeping a positive attitude), and concern for relatives and families, above having their pain and discomfort controlled at the end-of-life. This may reflect the strong sense of community within families that is still found in many parts of Kenya, and the importance of community above self [37-41] stemming from a different philosophical viewpoint of ‘individuals’ and the ‘community’ from that of the West. This suggests a fundamental difference in people’s priorities comparing to European countries [22-24,33,42], which should be reflected in the way end-of-life care is planned and provided in Kenya.

Yet, whilst keeping a positive attitude and family items were prioritised over pain management, pain was still seen as a problem of great concern, along with family and personal psychological distress. Pain is often one of the most feared symptoms in end-of-life care [43,44], and whilst much of the literature is based on a western perspective [45,46], previous African studies in palliative care settings found that pain is a major concern [26,34,47,48]. This study, within the general population, shows that anticipation of pain at the end-of-life is also an important issue. Effective pain management at the end-of-life is a relatively new concept and still not a reality for most patients in need in this country, with only an estimated 3% of people who need it able to access it, therefore work is being undertaken to increase accessibility and availability of analgesics, such as oral morphine [35]. It is interesting to compare these African priorities with European findings [23,24,33]. In the European arm of the study, pain was seen as the greatest concern of respondents across all seven countries surveyed, alongside that of keeping a positive attitude (both 36%), followed by relatives/friends not being worried or distressed (19%) and practical matters resolved (9%).

In Nairobi around three in five respondents prioritised improving quality of life over extending life, with one third not wanting to know they have limited time left. In relation to European findings, in all countries the highest priority was for improving the quality of life for the time left. This ranged from 57% in Italy to 81% in Spain. Only a small proportion wanted to extend life – ranging from 6% in Flanders to 2% in England [49], whereas in Nairobi this was considerably higher at 23.9%. Thus when planning for palliative care services in Kenya, whilst the focus is similar to that seen in Europe, and the majority have similar priorities, it is important to consider the larger group in Kenya who prioritized extending life over quality of life. It is necessary to study this group further in order to ascertain whether this is a difference that can be generalized or attributed to the younger sample, or to differences in the cause of death in Kenya, e.g. greater proportion dying from HIV [50].

In relation to decision-making about end-of-life care, less than half (47.8%) said they would want to be involved in decisions if they had the capacity to do so. This contrasts with what the European public wants. Findings showed that across seven European countries, 73.7% would want to be involved in decisions in a capacity scenario and 43.9% in an incapacity scenario, e.g. through living wills [33]. Preferences regarding the involvement of healthcare professionals appear to be similar in our survey in Nairobi. Within Europe (across the seven countries), in a scenario of capacity, 29.7% of the respondents wanted the doctor to be involved, and in a scenario of incapacity, 24.2% wanted doctor involvement [33]. These findings are critical in light of the importance of the family and the community within Kenyan culture, often above that of the individual [37,40,41] and in contrast to the culture often seen in Europe. Consequently, more attention should be given to ensure that palliative care service provision focuses on the individual and their family, with mechanisms to facilitate family decision-making on behalf of patients at the end-of-life, where appropriate [51].

In terms of place of death, international evidence shows the majority of people prefer to die at home, not in hospital [30,42,52]. In Nairobi we found that home was indeed the most common preferred place of death (51.1%) but also the least preferred place of death (23.7%), with 21.0% also stating their least preferred place of death being in the home of friends or family. Hospital was the second most preferred place to die, by around a quarter of the respondents. Although the preference to die at home is common to the findings of the PRISMA survey in Europe, a preference for hospital was much higher in Kenya (6.6% across seven European countries) [22]. It also differs from the results reported by Uwimana and Struthers [25] in Rwanda, who found that out of 250 people living with HIV/AIDS, 67% indicated they would prefer to be looked after in hospital during the terminal phase of illness, while 26% indicated they would prefer to be looked after at home. Whilst the study in Rwanda was undertaken not long after the civil war in the country and home may not have been seen as a ‘safe environment’, similarities exist in Kenya, following the post election violence in 2009. Another key finding from our survey in Nairobi is the percentage of people who preferred to die in a hospice or a palliative care unit, which is slightly lower but similar to that in Europe (15.6% compared to 19.7% in Europe [22]). This suggests that it is important to provide a variety of models of palliative care services and that people’s concerns with staying at home (as suggested by findings about the least preferred place of death) need to be better understood and addressed, whilst inpatient services may need to be developed more, if preferences are to be met. It is possible that in a collectivist society, such as Kenya, with a community focus, you may be more likely to want to be at home (=most preferred) but also we know there is little home palliative care coverage and people do not want to burden family and resources are low so there is a significant cost (=least preferred). Therefore, our findings support the need for a comprehensive strategy of palliative care provision in Kenya which takes into account individual preferences and allows for diversity [53].

Whilst this is an important study in beginning to identify preferences and priorities for end-of-life care in Africa, it has a number of limitations. The results show the preferences of a relatively young and healthy sample, which may not represent older and less healthy groups. However, as in Europe [22], we found that people’s experiences of serious illness, death and dying had little impact on their preferences and priorities for end-of-life care. The study was limited to an urban setting and specific areas of Nairobi, and whilst this was needed to take the safety of researchers into account, it could introduce bias into the sampling as the preferences for people from rural areas may vary from those from urban areas. Likewise, the days, language and times of the day in which the street survey was conducted may also have introduced bias, through not targeting those at work, and possibly those with a higher education level, who may be more informed about the healthcare services available at the end-of-life and who speak English. It is also important to note the fact that we were able to explain only a very small part of the variation in findings, with few socio-demographic differences detected, and in future studies more variables would be added including marital status, number of children and number of people living in the household. Hence the ability to discriminate groups who may have different preferences and priorities for end-of-life care is limited. This requires further research with a view to inform how clinicians can provide an individualised approach, assessing and addressing their preferences and priorities for end-of-life care.

## Conclusion

This is the first population-based survey on public preferences and priorities for end-of-life care in Africa. Psycho-social domains were seen to be of greatest importance to members of the general public, and pain control was a top priority, although access to pain management is inadequate. The survey shows what the majority wants (e.g. to be informed, to die at home) but also reveals variations that are not yet fully understood and important differences compared to European citizens (e.g. stronger importance of family, larger minorities of people who prefer to extend life and die in hospital). The findings prompt the development of palliative care services in Kenya to ensure a model of care that allows for both patient and family decision-making and provides care in the different settings (e.g. at home, in a hospice or a hospital) if citizens’ preferences and priorities for end-of-life care are to be met.

## Competing interests

The authors declare that they have no competing interests.

## Authors’ contributions

JD was the PI for the study, and a member of work package 2, contributed to the design and administration of the questionnaire, with special interest in Africa, developed the protocol, oversaw data collection and drafted the paper. BG is lead for work package 2 and led the questionnaire design for the European and African studies, analysed the results and assisted in revising the paper. NG and GM were research assistants involved in data collection and reviewed the paper. RAP, FNM-P, BD were involved in the analysis and review of the paper. IJH and RH are the Scientific Directors of PRISMA, co-obtained funding, contributed to the design and administration of the questionnaire, and revising the paper. All authors read and approved the final manuscript.

## Pre-publication history

The pre-publication history for this paper can be accessed here:

http://www.biomedcentral.com/1472-684X/13/4/prepub
